# Chinese heritage language motivation: a study of motivation development in a multicultural context

**DOI:** 10.3389/fpsyg.2024.1452547

**Published:** 2024-10-29

**Authors:** Xiaohong Wen

**Affiliations:** Department of Modern and Classical Langauges, University of Houston, Houston, TX, United States

**Keywords:** heritage language, cultural identity, contextual interactions, the anti-ought-to self, Chinese community, complex dynamic systems theory, language learning motivation

## Abstract

This study investigated motivation of Chinese heritage language (CHL) learners with diverse Chinese language backgrounds at an American university. Using a mixed-methods design, it examined the factors that motivated CHL learners to enroll in Chinese courses and continue their studies. The Study explored interactions that enhanced the learning experience and self identity development. A survey was conducted, followed by individual interviews. The results identified five motivation factors significantly correlated to the ideal L2 self, which functions as an anchor. The ideal L2 self was the strongest predictor of intended effort via positive attitude. The classroom-related experience was another significant predictor of intended effort. Interview data highlighted the dynamic interplay between sociocultural contexts and learner-environment interactions which provokes motivation development and strengthens identity reconstruction and future self-guides. In the process, the learner continuously constructs and consolidates the identity as “Chinese” relating to family and culture. Lastly, the anti-ought-to L2 self, characterized by reactions to “others,” emerged in dynamic interactions between learners and contexts. This motive inspired the learner to continuously develop the possible self and gain positive learning experiences.

## Introduction

Recent research on second language (L2) acquisition has adopted the framework of complex dynamic systems theory (CDST), shifting focus from the macro perspective to more specific learning settings where the motivation unfolds in a fluid and changing manner (Dörnyei, 2021; Dörnyei et al., 2015). Larsen-Freeman (2018) contends that the nature of L2 learning is “emergent from and dynamically interconnected with the environment” (p. 59). Ushioda’s (2009) Person-in-Context Relational View of Motivation highlights the agent role of learners by taking into account the fact that learners are located in particular sociocultural contexts. Along the same lines, Dörnyei’s (2009) L2 Motivational Self System links motivation to “the individual’s personal ‘core,’” (p. 9), emphasizing the learner’s ideal L2 self in dynamic interactions with context. In this process, learners negotiate their identities and visions of future selves to achieve language proficiency. This study, drawing upon the CDST framework and the L2 Motivational Self System, investigates the complexities of Chinese heritage language (CHL) learners’ motivation.

Although a good number of studies have applied the CDST framework and L2 Motivational Self System to diverse contexts worldwide (e.g., Csizér and Kormos, 2009; McEown et al., 2017; Papi, 2010; Yashima et al., 2017), most research focused on English. Dörnyei and Al-Hoorie (2017) also noted that theoretical paradigms developed for L2 learning motivation have been largely based on English studies. With 350 languages spoken in the USA (American Academy of Arts and Sciences, 2024), it is critical to extend the arena to include “languages other than English” (LOTEs). Different data sources from various learning contexts and language settings may affect research findings (Thompson and Vásquez, 2015). This study opts for a context of Chinese as a heritage language. Research on heritage learners’ motivation is limited, particularly in the CHL context (Xiao and Wong, 2014; Xie, 2014; Wen, 2011). CHL learners are an under-researched group whose motivation and identity development deserve much more attention.

## Literature review

### The L2 motivational self system and CDST

Dörnyei (2005, 2009) contends that motivation and a learner’s self-identity are shaped through ongoing situated processes with imagery of an ideal future self. A learner is highly motivated if the person envisions a possible future self and is able to take actions to reduce the discrepancy between the current and future self. The L2 Motivational Self System hinges on three key components. First, the Ideal L2 Self is the L2-specific facet of one’s “ideal self,” encompassing aspirations one would desire to achieve (Dörnyei, 2009, p. 29). It is powerful, as it generates strong future self-vision to inspire learners toward the goal. Second, the Ought-to L2 Self refers to the attributes one believes one ought to possess to meet familial and societal expectations. This preventive tactic is related to safety and social obligations. Both the ideal self and the ought-to self are conceptualized as future self-guides, influencing the actions taken in the learning process.

Third, the L2 Learning Experience encompasses situated “executive” motives related to the immediate learning environment. It is often referred to as the attitude toward the learning situation, such as enjoyment derived from the learning process (Yashima et al., 2017). Positive learning attitudes and experiences directly affect the formation of images of future selves, and vice versa (Csizér and Kormos, 2009; Kormos and Csizér, 2008; You and Dörnyei, 2016; Wen, 2013, 2022; Wen and Piao, 2020).

The initiation, development, and sustainment of motivation have been central themes not only in second language but also in heritage language motivation research. Learning an additional language, including one’s heritage language, is a part of the identity that reflects how one envisions oneself. Heritage learners’ complex socio-cultural environments shape their motivation and learning effort. Motivation, as a process and a situated construct, fluctuates over time and varies based on interactions with contextual variables during learning. For CHL learners, their learning experience is constructed in the process of understanding their own cultural heritage and their culture-related identity in addition to the learning environment (Comanaru and Noels, 2009; Pérez-Milans, 2021; Wen, 2011). This study, adopting the CDST’s holistic approach, not only measures motivation at a point in time and the relationships among motivation components, but also examines CHL learners’ identity emergence and development in response to complex cultural processes, drawing insights from the interview data.

### The anti-ought-to self and CHL motivation

One of the aims of this study is to examine the anti-ought-to self as proposed by Thompson and her associates (Thompson, 2017; Thompson and Vásquez, 2015; Liu and Thompson, 2018). Thompson and Vásquez (2015) postulate that what seems to be absent in the L2 Motivational Self System is the dynamic relationship between the learner and the context, i.e., the learner, as an agent, may have psychological reactance against societal or others’ expectations. Their concept of the “anti-ought-to self” centers on the interplay between the “I” and the “other” (cf. Higgins, 1987 self-discrepancy theory). Similar to the ideal self and the ought-to self, the anti-ought-to self functions as a personal future self-guide motivating learners to attain the opposite of social expectations, e.g., as observed, taking Mandarin courses not because of expectations from family members (the ought-to self), but an internal drive to embrace challenges (the anti-ought-to self). Thompson (2017, p. 39) emphasizes the importance of the learner’s role, arguing that “incorporating the anti-ought-to self into the L2MSS would allow for the type of future self that defines the learner as the prevailing force in the language learning process.”

Although limited in quantity, research has applied Dörnyei’s (2009) L2 Motivational Self System, integrativeness, and instrumentality to Chinese heritage and nonheritage languages in various dimensions. Studies (Comanaru and Noels, 2009; Lu and Li, 2008; Xie, 2014, Wen, 2011) have investigated ethnic heritage-related motivation with large samples of CHL learners. Findings indicate that learning one’s own heritage language is an integral part of self-identity for HL learners. Chinese ethnicity and heritage play a central role in shaping their sense of self. Furthermore, heritage learners feel more pressure to learn Chinese than non-heritage learners, either because of social/family pressures or because of a self-imposed feeling that they ought to learn the language due to their sense of belonging and community connection (Comanaru and Noels, 2009; Wen, 1997, 2011). CHL learners clearly experience social and psychological complexity in their motivation and in their HL studies.

Research has also analyzed the similarities and differences within the heritage group. HL learners can be further categorized into two distinct subgroups: a heritage language group who speak or at least understand the HL at home, and a heritage culture group who have little access to the HL at home but are culturally connected and motivated (Van Deusen-Scholl, 2003; Wen, 2011). The two subgroups of HL learners are more alike than different when compared to the non-heritage group (Comanaru and Noels, 2009; Rueda and Chen, 2005; Wen, 2011). They are more oriented to the ought-to L2 self, complying with family and social expectations, than the non-heritage group. However, few studies have explored the concept of rebellious motive with CHL learners. Previous research (Liu and Thompson, 2018; Thompson, 2017; Thompson and Vásquez, 2015; Wen, 2022) investigated the anti-ought-to self on various languages, excluding the heritage language. Wen’s (2022) study focused on L2 Chinese learners from multiple ethnic and language backgrounds. Factor analysis revealed that the construct of anti-ought-to self was a significant predictor for intended learning effort. It significantly correlated with all the motivation factors in the study, functioning as an intrinsic motivation featuring the active agent role. The current study, focusing exclusively on CHL learners, investigates motivation including the anti-ought-to self. It is of interest to examine whether the CHL group encounters societal and cultural pressures while learning one’s own heritage language; if so, what may provoke the desire to do the opposite of the ought-to self in the learning process?

### Chinese heritage language learners

CHL learners may speak their heritage language at an early age. They may experience language loss once they start attending school, where English is the dominant language (He and Xiao, 2008). Even if they continue to speak their heritage language, their linguistic development is restricted because of insufficient input. When entering college, they have an opportunity to take courses in their heritage language. Research on their heritage motivation, however, is scarce. This is not only because research in heritage languages in general is a new field, but also because most of the attention has been given to English as an L2 or foreign language. The increasing diversity of languages in the U.S. has highlighted the importance of HL education, in which the CHL plays a vital role. According to the U.S. 2018 Census, the ethnic Chinese population increased 104.1% from 1980 to 1990 and 50.1% from 2010 to 2017 (Wikipedia, 2023). In 2018, China replaced Mexico as the country sourcing the most immigrants in the U.S. foreign-born population (Echeverria-Estrada and Batalova, 2020). The rapidly increasing number of CHL learners is evident in heritage and immersion schools, K-12 Chinese courses, and college Chinese programs over the past two decades.

CHL learners share linguistic, cultural, and emotional characteristics (Comanaru and Noels, 2009; Valdés, 2001; Xie, 2014; Wen, 2011, 2022). Their linguistic skills vary to a large degree (Carreira and Kagan, 2018; He, 2008). Because of family and community exposure, their oral abilities are generally stronger than their literacy skills. Literacy competence, however, is emphasized in Chinese communities largely because the written form is a unifying symbol shared by various Chinese “dialects.” In comparison with nonheritage learners, HL learners’ stronger listening skills enhance their ability to access media. HL learners have an emotional connection to their HLs. They identify themselves with their heritage culture, learning the language that is a part of the culture and conveys it (Comanaru and Noels, 2009; Ganassin, 2020; Wen, 2011). Furthermore, they are interested in “how Chinese culture could be meaningful for their family histories and their own identities” (Ganassin, 2020, p. 160).

Defining CHL is further disputatious because of the term *Chinese*. The problem with referring to Chinese as one single language is that it neglects the linguistic diversity of Chinese languages (DeFrancis, 1984). *Chinese* encompasses a variety of languages that are mutually unintelligible, due to historical connections and geographical distributions. CHL is not uniformly Mandarin, but includes language varieties such as Cantonese, Teochew, Hokkien, Hakka. However, “to call Chinese a family of languages is to suggest extralinguistic differences that in fact do not exist and to overlook the unique linguistic situation that exists in China.” (DeFrancis, 1984, p. 56). The present study, recognizing the dilemma, opts to identify the CHL learner as an individual who “sees Chinese with a particular family relevance” (He, 2006, p. 1), i.e., who has familial and cultural connections with their HL, with Mandarin as the focus, since it is the primary language taught in the U.S.A.

### This study

This study aims to address gaps in L2 motivation research across multiple dimensions. First, it expands the L2 Motivational Self System framework by including the anti-ought-to self, a potential motivator that emphasizes the active learner’s role in interactions with contextual variables. Second, it explores learning experience, a previously overlooked component that significantly influences learning effort (Dörnyei, 2019). Positive experiences contribute to learning attitudes and motivation development (Henry, 2017). Third, it broadens the research arena by focusing on CHL learners, a large and distinct group underrepresented in motivation research. Fourth, the study adopts a mixed-methods approach, using quantitative analysis to compare data and qualitative methods to explore the interactive and emerging nature of L2 Chinese motivation and identity. The study’s holistic analysis aims to provide a more comprehensive understanding of CHL motivation and HL acquisition.

This study also explores motivation power, the gap between intended and actual learning effort. Positive learning attitudes and experiences are linked to higher levels of learning engagement and committed effort. Motivation power is typically measured as the self-reported intention for effort, but there may be discrepancies between intended and actual effort. It is unclear whether intended effort actually reflects motivated behavior (Yashima et al., 2017). Papi (2010) called attention to this potential difference. This study aims to examine both intended and actual effort, comparing the actual effort of CHL learners in this study with that of CFL non-heritage learners in Wen’s (2022) study.

This study was guided by three research questions:

1. What motivation components encourage CHL learners to study their heritage language in an American university setting?
2. How are these factors interrelated in the context of CHL learning?
3. How do sociocultural factors and learner-environment interactions shape CHL learners’ motivation and identity development?

## Methodology

### Setting and participants

The study was conducted at a US university located in a multi-ethnic city with a large Chinatown. The university offers a B.A. degree and a minor in Chinese Studies. Participants consisted of 61 CHL students (mean age: 21.1) enrolled credit-bearing courses at all proficiency levels. All the participants had at least one Chinese relative. Many were multilinguals, speaking Chinese regional variants, and/or Spanish or Vietnamese, in addition to English while taking the Mandarin Chinese course. Most (90.1%) were American-born, and 72% were third-generation with their grandparents being the only non-English-speaking family members. Table 1 provides detailed demographic information about the participants.

**Table 1:** Participants’ demographics.

Information	Specifications	Number
Gender	Male	24
	Female	37
Proficiency levels	Elementary	25
	Intermediate	18
	Advanced	18
Ethnic	Chinese American & Chinese	42
Backgrounds	Mixed	19
Current home	Chinese	16
Language	English	11
	Mixed Chinese & English	34
First language	English	36
	Chinese regional variants	20
	Spanish	3
	Others	2
Chinese-speaking	Parents	15
Family members	Grandparents	44
	Relatives	2
Previous learning	High school	15
Chinese	Chinese school	27
	None	19
Language requirements	Yes	20
	No	41

Among 61 participants, 12 voluntarily participated in subsequent individual interviews. All interviewees were multilingual in English, Spanish, Cantonese, and/or Asian languages while taking Mandarin courses.

### Instrument

The survey instrument consisted of three sections. The first section had 16 items on the learner’s demographics. The second section was a motivation scale, consisting of 25 seven-point Likert-scale items assessing the ideal L2 self, the ought-to L2 self, the anti-ought-to self, attitudes and experiences in learning L2 Chinese, instrumentality-promotion, and intended effort. These items were adopted from previous studies (Liu and Thompson, 2018; Wen, 2022; Yashima et al., 2017) with minor adjustments to suit the context of this study. Item examples include: the ideal L2 self “Studying Chinese is important to me to gain the approval of my peers/teachers/family/boss”; the anti-ought-to self “I am studying Chinese because it is something different or unique”; learning experience “I find learning Chinese really interesting”; instrumentality “Studying Chinese is important to me to achieve my academic goals”; and intended effort “If my teacher gave the class an optional assignment, I would certainly volunteer to do it.” By including items from the previous studies with some revisions pertinent to learning CHL in the USA, the survey aimed to provide a more valid description of motivation.

The third section of the survey focused on motivated behavior, measuring the weekly time spent on related after-class learning activities. The items were in three clusters: (1) Chinese course-related work, (2) Cultural and language contact, and (3) Interpersonal communication. A pilot survey was conducted with 15 CHL students, who were asked to list their weekly after-class Chinese learning activities. The most frequently mentioned activities were identified to create the After-class Activity Engagement form Table 2.

**Table 2:** After-class activity engagement.

Chinese course-related work	Cultural and language contact: Learning via Media	Interpersonal communication
Do Chinese course related work	Listen to radio, podcasts, and audio/visual streams in Chinese	Speak Chinese with people in Chinatown
Do extra/unrequired course related work for the course	Conversing, reading, and writing messages in Chinese via social media (email, Facebook, cell phone, etc.)	Speak Chinese with Chinese friends
	View movies, TV, videos in Chinese	

In addition, semi-structured interviews were conducted. Interview questions were developed based on the study’s goals. Although the questions were pre-planned, new topics were discussed as they arose during the interviews. The interview questions included the factors that influenced the choice of Chinese language, learning strategies that particularly sustain learning, stories of overcoming learning difficulties as an CHL learner, and the interaction between future self-guides and the learners’ engagement in the learning process. An inductive approach was used for data coding and analysis. Prominent themes identified in the transcripts were analyzed to understand the complexities of CHL learner motivation.

### Procedure

The survey was announced in the spring semester. CHL students voluntarily filled out the survey on a webpage. Two weeks after the questionnaire was administered, individual virtual interviews were conducted via Zoom in a conversational manner. Each interview lasted an average of 50 min and was recorded with the interviewee’s consent.

### Data analysis

This study first investigated motivation components. Factor analysis identified underlying motivation factors. Additionally, interview analysis further identified deeply rooted motivational thoughts, sociocultural identities, and nuanced learning experiences. Correlation analysis examined the associations among these factors, including after-class learning engagement. Regression procedures were performed to explore predictors of intended effort via positive attitude. Furthermore, the study compared the CHL group in this study and the non-heritage group (Wen, 2022) in terms of after-class activity engagement, as both studies used the same instrument. Finally, the study analyzed interactions between motivation development, identity negotiation, and learning experience. Interview data themes were examined to triangulate quantitative findings. Specific areas of focus included learner’s encounters with contextual obstacles, their adopted strategies, and how they overcame difficulties to sustain learning.

## Results

### Factor analysis to explore CHL motivation constructs

Exploratory factor analysis (EFA) was conducted to detect the motivation constructs. The Principal Component Analysis and Varimax method were performed. The Kaiser-Meyer-Olkin value was 0.77, indicating an adequate sample size. Although eigenvalues of all 25 items were greater than one, two items related to the anti-ought-to self (#46 “I would like to reach a high proficiency in Chinese, despite others telling me that it will be very difficult.” and #51 “Learning Chinese builds up my self-esteem because Chinese is considered to be difficult to learn.”) did not load. In the second run of EFA, the remaining 23 items all loaded, extracting five factors accounting for 71.78% of the total variance. The factor loadings (Table A1) confirmed the validity of the survey instrument. Table 3 displays descriptive statistics, Cronbach’s internal coefficient, and the amount of the total variance each factor explained.

**Table 3:** Descriptive statistics, internal consistency, and variance explained.

Factor #	Motivation factors	Mean	SD	Min	Max	Cronbach’s α	Total variance explained
1	Intended effort via positive attitude	5.22	1.15	2.14	7.00	0.87	39.37%
2	Instrumentality- promotion	4.80	1.55	1.80	7.00	0.89	10.98%
3	Ought-to self	4.33	1.73	1.00	7.00	0.84	9.17%
4	Classroom-related experience	5.74	1.09	2.33	7.00	0.81	6.64%
5	Ideal L2 self	5.26	1.27	1.50	7.00	0.80	5.62%

Factor 1, *Intended effort* via *positive attitude,* refers to intended effort from positive learning attitude (cf., Yashima et al., 2017). Items include the intended effort: “I am prepared to expend a lot of effort in learning Chinese” and attitudes derived from positive learning experience: “I find learning Chinese really interesting” and “I enjoy a challenge with regard to Chinese learning.” Dörnyei (2019, p. 1) defines learning experience as “the perceived quality of the learners’ engagement with various aspects of the language learning process.” This factor, intertwining intended effort and positive learning experience/attitude, fits into the comprehensive concept of “learning experience.”

Factor 2 *Instrumentality-promotion* relates to the imagined future self for global career development or academic advancement. The factor reveals the aspiration to use the language to achieve certain goals. Items include “studying Chinese is important to me because with Chinese I can work globally,” and “to achieve my career goal.” These desires align with a visualized successful future self, emphasizing accomplishments, hopes, and aspirations (Higgins, 1998, p. 10).

Factor 3 *The ought-to L2 self*, refers to accommodating external expectations from family and friends. Unlike the ideal L2 self, which focuses on intrinsic desires, this factor emphasizes perceived obligations. Items include “Studying Chinese is important to me because people I respect think I should study it.”

Factor 4 *Classroom-related experience* is closely related to the immediate learning context: “I like the atmosphere of my Chinese classes,” “I like the teaching style of my Chinese language teacher,” and “I find the textbooks and learning materials to be useful.” The classroom is a complex and dynamic environment that can motivate or demotivate students.

Factor 5 *The ideal L2 self* emphasizes the use of language for daily communication and views Chinese language competence as an integral part of one’s self-identity. Items include “I can imagine myself as someone who is able to speak Chinese,” “living in Chinese-speaking areas and using Chinese effectively to communicate with the locals,” and “reading and writing emails in Chinese fluently.” This future vision motivates learners to achieve their language goals and become proficient Chinese language users.

*The anti-ought-to self* did not emerge as a separate factor in the EFA procedure. Originally, there were four items. Two items did not load; the remaining two (#31, I am studying Chinese because it is something different or unique, and #36, I enjoy the challenge of learning Chinese.) loaded to Factor 1, *Intended effort* via *positive attitude.* This finding is inconsistent with previous research (Liu and Thompson, 2018; Thompson and Liu, 2018; Wen, 2022), which identified the anti-ought-to self as a distinct motivational factor in L2 learning. However, unlike these studies, the current research focuses exclusively on heritage language learners. Given that the anti-ought-to self was initially proposed based on qualitative findings (Thompson, 2017; Thompson and Vásquez, 2015), it is crucial to analyze the interview narratives of this study.

### Correlation analyses to examine the relationships among the motivation components

Pearson correlation coefficients were used to analyze the relationships between factors. Factor 1, *Intended Effort* via *Positive Attitude*, significantly correlated with all factors (*p* ≤ 0.01, two-tailed), except for *the ought-to L2 self*. Similarly, Factor 4, *Classroom-Related Experience*, correlated significantly with all factors (p ≤ 0.01, two-tailed), except for *the ought-to L2 self*. These findings suggest that *the ought-to L2 self* in this study has minimal influence on classroom-related learning experiences and attitudes toward effort. Factor 2, *Instrumentality-Promotion,* significantly correlated with all factors, indicating its potential as a primary motivator. Importantly, *Instrumentality-promotion* and *the ideal L2 self* significantly correlated (*r* = 0.657, *p* ≤ 0.01, 2-tailed), suggesting that *instrumentality-promotion* may indeed be a subcomponent of *the ideal L2 self* as Dörnyei (2009) proposed. It is worth noting that factor 5, *the Ideal L2 self,* significantly correlated with all motivation factors, highlighting its strong link to overall learner motivation and its harmony with the *ought-to L2 self* (Dörnyei, 2009). Two after-class activity engagements, *Intercultural and language contact* via *media* and *Interpersonal communication,* were significantly correlated, indicating that students who enjoy using media to learn Chinese culture and language are more likely to engage in interpersonal communication in Chinese. The results of the Pearson correlation coefficient are presented in Table A2.

Stepwise regression was performed to identify the predictors of *intended effort* via *positive attitude*. The first independent factor entered into the procedure was *the Ideal L2 Self,* followed by *Classroom-related experience*. The remaining factors did not enter the procedure. These two factors accounted for 44.4% of the variance in *intended effort* via *positive attitude*. The *ideal L2 self* in this study reconfirmed its high motivational magnitude and the strongest predicting power (*B* = 0.504), followed by *Classroom-related experience* (*B* = 0.423). The results demonstrated that CHL learners motivated by *the ideal L2 self* and *Classroom-related experience* are more likely to exert consistent effort in their learning.

Table 4 presents the model summary; Table 5 shows the coefficients of stepwise regression of *intended effort* via *positive attitude*.

**Table 4:** Predictor model and ANOVA: stepwise regression of intended effort.

Model	*R*	*R*^2^	*F*	*p*
1	0.556	0.309	24.561	<0.001
2	0.666	0.444	21.576	<0.001

**Table 5:** Stepwise regression of intended effort via positive attitude.

Model	Predictors	Unstandardized coefficients B	Standardized coefficients Beta β	*t*	*p*
1	Ideal L2 self	0.504	0.556	4.956	<0.001
2	Ideal L2 self	0.364	0.401	3.647	<0.001
	Classroom related learning experience	0.423	0.399	3.628	<0.001

The same regression procedures were performed on three after-class activity engagements with each as the criterion and motivation factors as predictors. Several marginally significant relationships were found. Factor 4 *Classroom-related experience* was a significant predictor (*p* ≤ 0.05) of *course-related engagement: F*(1,59) = 4.810, *p* = 0.033, *R*^2^ = 0.080. Factor 2 *Instrumentality-promotion* was a significant predictor of *interpersonal communication: F*(1,59) = 13.076, *p* < 0.001, *R*^2^ = 0.438; and a significant predictor of *intercultural and language contact* via *media: F*(1,59) = 5.125, *p* = 0.028, *R*^2^ = 0.085. These regression and correlation analyses between the motivation factors and actual after-class engagements suggest that these two constructs may be distinct, i.e., intended effort may differ from students’ actual effort.

This study compared the CHL and non-heritage groups’ motivated behavior, using the same instrument for measuring after-class activity engagement as Wen (2022). Welch’s t-test for unequal sizes and variances was employed. The results revealed significant differences in Chinese Course-related Work (*t*(67) = 4.30, *p* < 0.000) and Interpersonal Communication (*t*(116) = 3.64, *p* < 0.000) between the two groups. The CHL group demonstrated significantly higher effort in course-related work (*M* = 3.85) compared to the non-heritage group (*M* = 1.47). In terms of Interpersonal Communication, the non-heritage group (*M* = 3.59) engaged in significantly more interactions than the CHL group (*M* = 1.88). Although there was no significant difference in cultural and language contact via media, the CHL group used media more frequently (*M* = 4.44) than the non-heritage group (*M* = 3.20).

### Interview data

Interview narratives represented participants’ visions for their possible selves and desired self-identity in relation to their Chinese language ability and sociocultural integrativeness, as well as their learning experiences both within and beyond the classroom. The interview analysis in this study revealed the development of motivation in context and the presence of the anti-ought-to self motive. Importantly, the data provided nuanced insights into the interactions between motivation and contextual learning experiences. The excerpts quoted below use participants’ pseudonyms followed by their proficiency levels.

### Motivation development in context

The analysis addressed all three research questions, with a focus on the third question: the process of motivation development and identity construction through individual-contextual interactions. This included the reasons students initially chose to learn Chinese, their cultural inspirations, and the motivation that sustained their learning process.

Bennett (Advanced) had a language background in Cantonese, double majoring in Speech Pathology and Chinese Studies. In her first year at the college, she served as a translator for her department’s clinic patients who could only speak Cantonese. She was surprised to find that there were only two people in her entire department who could speak Cantonese. The usefulness of the language (the ought-to L2 self) motivated her to take Chinese courses. Working as a research assistant and growing up in a Chinese family, she played several roles and constructed her identity as a translator for Cantonese-speaking patients, as a Chinese community messenger for speech impairments, and as a trained professional. In the process, she realized that there has existed a cultural stigma surrounding speech and language disorders among Asians in her community. She wanted to be the one who could raise awareness of the disorder symptoms (the anti-ought-to self), and become the one who could reach out to help in their languages (her ideal self):

**Excerpt 1**

But I definitely want people to know that there is a hand that can reach out to them and help them if they have difficulties in speech and language, for them not to feel shame, and also to become a speech therapist who hopefully has a bilingual license … [I] am able to help individuals in multiple languages and understand the culture that they are coming from.

Her clear vision of her multi-lingual competence, her strong desire for calling attention to speech disorders in the Asian community, and instrumentality of the language motivated her to enroll in Chinese courses. The vivid image of being that “*helping hand”* was the inspiring self-identity. Through her experiences, she further developed a concrete image of her ideal self: to “become a bilingually certified and multi-culturally certified speech pathologist.” She continuously negotiated and constructed her identities while working with her Chinese community, as her Chinese proficiency and the community needs increased.

In her senior year, she expanded her identities to include Mandarin Chinese tutor. Because of her high Chinese proficiency, she was selected to be a student tutor, helping her peers with their learning difficulties. This tutoring role further fostered her CHL motivation. She constructed her “teaching-learning” moments during her tutoring, as noted in her retrospective accounts:

**Excerpt 2**

[Sitting next to classmates], hearing and watching their learning process also going through my own learning process alongside them allowed me to have a more robust view of the Chinese language and about learning Chinese.

Interacting with her classmates further consolidated her motivation with accompanying procedural strategies to improve her learning. Two months before her graduation, she was admitted to a graduate school to pursue her future image and identity: The “*reaching out multilingual hand*” for people who had speech symptoms, particularly among Chinese and Asians.

Her Chinese language motivation, first triggered by the usefulness of the language (the ought-to L2 self as a Cantonese translator), developed over time toward her ideal L2 self. Her personal calling and identity (the possible self as a multilingual speech pathologist) and her professional sense of raising awareness about language and speech disorders against the stigmatization among Asians (the anti-ought-self) functioned in harmony, inspiring her to succeed in both Mandarin Chinese and speech pathology studies.

Claire (Advanced) had a mixed ethnic background with her mother being Chinese and father Vietnamese. Both parents spoke English. Her grandparents spoke Cantonese only. Like many CHL learners, her future vision instinctively identified her with the Chinese culture and being able to speak Chinese with native speakers (NSs), particularly her relatives and grandparents. The ought-to L2 self also played an important role for at least two reasons. First, she wanted to comply with societal expectations. People expected her to speak the language because of her Chinese appearance. She felt embarrassed that she could not. Second, she needed to fulfill her foreign language course requirements. Both self-guides (the ideal and ought-to L2 selves) impacted her learning choices at the beginning:

**Excerpt 3**

When people look at me, they expect me to speak Chinese. So I feel like I should speak Chinese, and to talk to my mom and my grandma, and my relatives. I have to learn how to speak with them. … When I got to college, they [her college] told me I had to take a second language. I decided to take Chinese, because even though I know some words in Chinese, there are gaps in my knowledge. I need to fill them in.

Claire’s desire for her identity, “highly proficient,” was clear and concrete in terms of her language goals (Excerpts 3, 4). Her family and her sociocultural identity were a powerful internalized force (cf., Ushioda, 2009). Such motivators energized her to pursue her goals of carrying on substantial conversation with her family members:

**Excerpt 4**

I’m motivated by my family, ‘cause I want to be able to talk to them, especially with relatives who do not speak English, they speak only Chinese. … I want to get past the [stage of] ‘how is the weather’ ‘School is ok.’ I want to pass the really simple questions and go to deeper discussion.

In her Chinese classes, however, she encountered the opinion of ‘others’ who assumed that learning Chinese was easy for her because of her CHL background. She had no Mandarin background, and her Cantonese knowledge was minimal. Confronting the unfair expectations of others, she was determined to study well and strove to manage external pressures positively. She was elected president of a student association. One of her agendas, as the president, was to raise awareness of unfair assumptions toward the students who were American born and “look Chinese but had little Chinese language background.” Clair’s motivation then developed far beyond her original ought-to L2 self. Her identity as a CHL learner with little language background became consolidated when she played a sociocultural role as the student organization’s president, trying to eliminate bias toward American-born Chinese language learners. Her motivation interacted with her CHL learning goals, attitudes, and her classmates, which positively supported her as she negotiated her identities and her continuing Chinese studies.

### The emergence of the anti-ought-to self in the CHL context

Research (Thompson and Vásquez, 2015; Thompson, 2017) demonstrates that the emergence of the anti-ought-to self is much influenced by context. Indeed, the anti-ought-to self emerged in this study when learners interacted with their environment in a rebellious way. It appears as a result of interactions with parents, teachers, and the availability of learning opportunities. Contextual pressures triggered learners’ deeply rooted desires and self-identity. They reacted to the situation out of their visions for possible future selves. Their motivation went through changes in the sociocultural process when their identities were renegotiated, and the anti-ought-to self was constructed. The following cases are merely examples from interview narratives.

For Bennett (Advanced), the anti-ought-to self emerged from her frustration in a HL classroom context, particularly her teacher at a Chinese school. Her parents sent her there because “my Chinese was just really bad.” She had a teacher who asked students to copy what she wrote on the chalkboard. The teacher was unsatisfied with Bennett’s slow-paced copying and yelled at her:

**Excerpt 5**

She would just go up to the chalkboard and write like a long list of Chinese characters. I have no idea what she was writing. And it was so fast and everybody was done, but for me or my sister, …, we had to stay back in class and copy somebody else’s notebook in order to copy those characters. The teacher was just mean and strict, yelling at me.

Bennett’s interactions with her teacher were humiliating, as she struggled to keep up with her peers and understand character meanings. Unlike the previous time when she asked her parents to stop sending her to Chinese school, this time her anti-ought-to self emerged and she decided to “show the teacher my ability.” Her motivation had changed in this formal instructional context. Her identity had been reconstructed. She said that by then she “grew up enough to learn Chinese well” (the ideal self). In addition, she did not to want to disappoint her parents anymore (her ought-to self). Her future self-guides led her to take action. She developed her procedural strategies in learning:

**Excerpt 6**

I just made up my mind that this teacher is so mean, but I wasn’t going to stay back in class …. I began to really, really practice, like looking at how she wrote the strokes, like I hated how boxy my characters were and how ugly they looked. …. I just kind of really did not want to be beaten down by this teacher. So I became better.

The anti-ought-to self was prominent in Bennett’s reaction to her teacher. Equally importantly, she was also inspired by her possible self, her strong identity, and the ought-to L2 self. Multiple motivators emerged harmoniously, effectively enhancing her psychological resistance to the humiliating situation.

Sarah (Elementary) had a mixed family, with her mother proficient in Chinese although she did not usually speak Chinese at home. Her father, a Caucasian, only spoke English. Her father requested that English should be the home language because he would like to participate in conversations. Sarah never had opportunities to receive formal Chinese instruction, either in Chinese school or high school. Her grandparents were her only source of Chinese language input during visits. She felt a deep obligation to learn the language (the ought-to self), not only for the sake of communicating with her grandparents, but also to understand Chinese culture as practiced in the street and social events (instrumentality-promotion focus). She wanted to read street signs and understand the lyrics of the songs to which groups of women danced daily in Chinese parks. She identified herself as Chinese, despite her mixed ethnic background, who loves Chinese culture and should be able to understand the language (the Ideal self and integrativeness). Her psychological reactance toward her “non-opportunity” environment emerged to promote her to teach herself. The magnitude of her motivation was evident in her daily learning. She “would write each character 50 times daily.”

Sarah was not alone. Chaoyun (Intermediate) was another example although their contexts were different. Chaoyun took Chinese courses as a result of her psychological reactance to her parents, who did not send her to Chinese schools while she watched her friends attending. She felt that she had missed an important opportunity to learn Chinese language. She was expecting to learn Chinese in high school but “Chinese wasn’t offered” there. She could speak simple Cantonese when she was young. Later, she realized that Mandarin was too important for her to miss. As she commented: “I regretted it a lot: the fact that I did not go to a Chinese school at a young age.” Her vision for her ideal L2 self was suppressed to the extent that she became confused (“that should not be the case”) about her self-identity because her Spanish was better than her Chinese:

**Excerpt 7**

I am learning Spanish and I have been learning Spanish for about 6 years …, I felt a bit embarrassed, cause I know Spanish better than I know Chinese, and I felt like that should not be the case.

She took a Chinese course immediately upon entering college, as a rebellious action to her previous environments, which denied her the formal learning opportunity. Her anti-ought-to self was mingled with her future ideal self, as well as the ought-to L2 self: to meet public expectations. There was an incident where the “public” was her relatives. She experienced embarrassment at a family reunion dinner, where everyone was speaking Chinese. One aunt asked her a few questions. She could not even understand the questions. She asked her cousins next to her to translate the questions into English, although she still could not answer the questions. As she commented:

**Excerpt 8**

…. So that moment of feeling so embarrassed, not being able to have a conversation with my relatives on that dinner table in front of everyone, I felt like, oh I have to learn Chinese!

This moment of frustration turned into the motivating point for her to become competent in Chinese, to fulfill her heritage language identity and her ideal self. It should be noted that such situational interactions with people (in many cases, family members) who cannot speak English were shared by almost all interviewees although the consequences varied with contexts and individual language abilities. The most frequent contexts included family reunion dinners, classroom interactions, events in the Chinese community, and encounters with a Chinese NS who could not speak English. These contexts strengthened the learner’s ideal L2 self, fostering learning experience and developing Chinese heritage identity.

## Discussion

Situated in the context of CHL, this study has examined all three key components of the L2 Motivational Self System and a closely related factor, the anti-ought-to L2 self. Both quantitative and qualitative data yielded a wealth of information about multiple motivation components perceived by participants as important to their learning. One finding distinctive for CHL learners is their strong inner desire to be identified with their heritage culture and to be highly competent in their HL, particularly when communicating with their family members. The ideal L2 self correlates significantly with all the motivation factors, suggesting its strong link to learners’ overall motivation, and its harmony with the ought-to L2 self. This factor, together with *Classroom-related experience,* accounted for 44.4% of the variance in *intended effort* via *positive attitude*.

Learning experience, the third component in the L2 Motivational Self System, evolved through interactions, particularly classroom related interactions. This study demonstrates that *Classroom-related experience* is vital for learners. The classroom, as a dynamic learning context, is where the participants meet with an encouraging atmosphere to interact with peers, complete learning tasks and activities, and find their fit to a variety of instructional techniques. Interviewees explicitly described the class activities that they immensely enjoyed, and from which they derived a strong sense of self-confidence. Moreover, these interactions generate further motivation that in turn enhances the classroom environment. It is worth noting that the instructor plays a critical role in fostering learners’ positive experience. It is the instructor who initiates the classroom tone, designs the class activities, and provides opportunities for learners to interact and develop their positive learning experience.

The comparisons between the CHLs in this study and the nonheritage group (Wen, 2022) in terms of their motivated behavior (After-class Activity Engagement) indicates that the CHL group invested significantly more effort in course related work, which may directly contribute to grades and course achievement. Although the CHL group spent more time engaging with media in Chinese, the difference was not statistically significant between the two groups, suggesting that the non-heritage group also used media in Chinese almost as much. The non-heritage group excelled in interpersonal communications, such as speaking with Chinese friends and people in Chinatown. This may be because the CHL group communicates more frequently with family members, resulting in less interaction with Chinese friends and people in Chinatown.

This study has captured complex interactions between the dynamic environment and individual motivation experience, self-regulatory strategies, and learning effort. The interview analysis particularly demonstrates that motivation emerges and develops in context, especially when the learner encounters unexpected incidents, positive or negative. Motivation actively interacts with learners’ ethnic identity, attitudes, and contextual factors, which enhances their learning experience to sustain learning. In addition to meeting challenges while persistently strengthening the ideal and ought-to L2 selves, CHL learners, inspired by the anti-ought-to self, react to external pressures. The anti-ought-to self generates a powerful future self-guide, encouraging learners to be active and autonomous. When confronting external pressures, they are not demotivated but develop a clear understanding of their strengths and weakness. Their motivation and identities develop as they apply their individual procedural strategies, derived from their future self-guides, to their learning.

It is interesting that the anti-ought-to self did not appear in the quantitative analysis but emerged in the interview data. The concept of the anti-ought-to self was first proposed in qualitative studies (Thompson, 2017; Thompson and Vásquez, 2015). However, quantitative research results have been inconsistent. Thompson and Liu (2018) found the anti-ought-to self in L2 English and Japanese, but not in French. They speculated that the anti-ought-to self emerges only when public opinion or controversy is involved with the language. In the context of CHL, society and communities generally expect CHL learners to acquire their heritage language, there does not seem to be “public opinion or controversy involved” at the macro level. However, at the micro level, in-depth individual interviews enable learners to reflect on their learning experiences and emotions, revealing subtle details and nuance that quantitative research may miss. Therefore, the CDST framework and the interview data used in this study are confirmed to be critically necessary.

The anti-ought-to self is still relevant to CHL even although learning CHL is congruent with the learner’s ethnic identity. The distinction between individual “self” and “others” is central to the notion of the anti-ought-to self (Thompson and Vásquez, 2015). In a broad sense, the term “others” refers not only to public opinions but also to contextual pressures that provoke learners to react and to “stand out” (Thompson, 2017). The desire to respond to contextual pressures is a deeply rooted future vision originating from the learner’s inner self. Its self-guide brings out effort and empowers action. Examples of psychological reactance in this study include: (1) aspirations and committed effort for Chinese language competence when confronting the absence of learning opportunities, (2) demonstrating ability in response to a teacher’s condescending attitude toward slow-paced learning, and (3) being the “reaching out multilingual hand” in the Asian community. In these contexts, the ideal L2 self and the anti-ought-to self are intertwined. Both are intrinsic, with the latter opposing contextual obstacles imposed upon learners. A learner’s desire to become the imagined future self initiates action as an active agent to achieve one’s own goals despite external opinions and environmental adversities.

The inconsistency in detecting the anti-ought-to self in CHL may be attributed to both the instrument and the participants. There may exist the anti-ought-to self with CHL, but the EFA conducted in this study may not have been sensitive enough to identify it. The items used may not be suitable for the CHL group. These items, adopted from Liu and Thompson (2017) and Wen (2022), were originally designed for non-heritage learners. The research context of Liu and Thompson (2017) was in China, focusing on L2 English. To accurately measure the anti-ought-to self in CHL, future research should develop more relevant survey items. This requires a careful conceptualization of the construct within the specific context of CHL acquisition.

The findings of this study have direct implications for L2 pedagogy. Effective instruction, as presented in the factor *Classroom-related experience,* should be tailored to learners’ individual needs to foster positive learning experiences and help construct a more successful future self. The key components of this factor: class atmosphere, teaching style, and learning materials, directly impact learning and enhance motivation development. In a dynamic classroom where the atmosphere is encouraging, instruction accommodates diverse learning styles, and learning materials are at the appropriate level, learning motivation thrives and cultural identity consolidates.

## Conclusion

This study adopted the CDST framework to investigate how CHL learners generated and developed their motivation. Exploratory factor analysis identified five motivation constructs: (1) *Intended effort* via *positive attitude,* (2) *Instrumentality-promotion,* (3) *the Ought-to L2 self*, (4) *Classroom-related experience*, and (5) *the Ideal L2 self*. *The ideal L2 self* functioned as an anchor, significantly correlating with all motivation factors and emerging as the strongest predictor of *intended effort* via *positive attitude*. Classroom-related experience was another significant predictor of intended effort. Interview data highlighted the dynamic interplay between sociocultural contexts and learner-environment interactions, which influenced motivation development, identity reconstruction, and future self-guides. Complex contextual interactions created pivotal moments for learners to construct positive learning experiences and respond to environmental challenges. The anti-ought-to self did not appear in the quantitative analysis but emerged in the qualitative analysis. The role of the anti-ought-to self across quantitative and qualitative methods warrants further investigation in future research. To accurately measure the anti-ought-to self in CHL, future studies should develop more relevant survey items, grounded in a careful conceptualization of the construct within the CHL context.

## Data Availability

The raw data supporting the conclusions of this article will be made available by the authors, without undue reservation.

## Appendix

**Table A1:** Results of factor loading.

Item#	Factor 1 loading	2	3	4	5
Factor 1: Intended effort via positive attitude					
56	0.867				
37	0.834				
55	0.825				
36	0.794				
57	0.656				
54	0.557				
31	0.486				
Factor 2: Instrumentality- promotion					
53		0.793			
44		0.748			
43		0.650			
38		0.582			
48		0.576			
Factor 3: Ought-to L2 self					
30			0.834		
50			0.796		
35			0.776		
45			0.767		
Factor 4: Classroom-related experience					
32				0.850	
47				0.796	
52				0.668	
Factor 5: Ideal L2 self					
33					0.792
49					0.754
39					0.679
29					0.436

**Table A2:** Pearson correlations of motivation factors and after-class activities (two-tailed).

Factor & After-class activities (AC)		*Intended effort via positive attitude*	Instrumentality Promotion	Ought-to self	Classroom-related *experience*	Ideal *L2* self	Course related engagement	Intercultural via media	Interpersonal communication
1	*Intended effort via positive attitude*	1.00	0.545**	0.256	0.554**	0.556**	0.005	0.239	0.090
2	*Instrumentality promotion*		1.00	0.346**	0.548**	0.657**	0.130	0.292*	0.438**
3	*Ought-to self*			1.00	0.086	0.336*	−0.034	0.074	0.179
4	*Classroom-related experience*				1.00	0.387**	0.283*	0.143	0.195
5	*Ideal L2 self*					1.00	0.011	0.228	0.325*
AC1	*Course-related engagement*						1.00	0.493**	0.489*
AC2	*Intercultural contact: via Media*							1.00	0.579**
AC3	*Interpersonal communication*								1.00

**Correlation is significant at the 0.01 level (two-tailed). *Correlation is significant at the 0.05 level (two-tailed).
